# A Portable Readout System for Biomarker Detection with Aptamer-Modified CMOS ISFET Array

**DOI:** 10.3390/s24103008

**Published:** 2024-05-09

**Authors:** Dmitriy Ryazantsev, Mark Shustinskiy, Andrey Sheshil, Alexey Titov, Vitaliy Grudtsov, Valerii Vechorko, Irakli Kitiashvili, Kirill Puchnin, Alexander Kuznetsov, Natalia Komarova

**Affiliations:** 1 Scientific-Manufacturing Complex Technological Centre, 1–7 Shokin Square, Zelenograd, Moscow 124498, Russia; 2 Institute of Nanotechnology of Microelectronics of the Russian Academy of Sciences, 32A Leninsky Prospekt, Moscow 119334, Russia; 3 Municipal Clinical Hospital No.15 Named after O.M. Filatov, 23 Veshnyakovskaya St, Moscow 111539, Russia

**Keywords:** ion-sensitive field effect transistor, aptamer, biosensor, troponin I, acute myocardial infarction, point-of-care testing

## Abstract

Biosensors based on ion-sensitive field effect transistors (ISFETs) combined with aptamers offer a promising and convenient solution for point-of-care testing applications due to the ability for fast and label-free detection of a wide range of biomarkers. Mobile and easy-to-use readout devices for the ISFET aptasensors would contribute to further development of the field. In this paper, the development of a portable PC-controlled device for detecting aptamer-target interactions using ISFETs is described. The device assembly allows selective modification of individual ISFETs with different oligonucleotides. Ta_2_O_5_-gated ISFET structures were optimized to minimize trapped charge and capacitive attenuation. Integrated CMOS readout circuits with linear transfer function were used to minimize the distortion of the original ISFET signal. An external analog signal digitizer with constant voltage and superimposed high-frequency sine wave reference voltage capabilities was designed to increase sensitivity when reading ISFET signals. The device performance was demonstrated with the aptamer-driven detection of troponin I in both reference voltage setting modes. The sine wave reference voltage measurement method reduced the level of drift over time and enabled a lowering of the minimum detectable analyte concentration. In this mode (constant voltage 2.4 V and 10 kHz 0.1Vp-p), the device allowed the detection of troponin I with a limit of detection of 3.27 ng/mL. Discrimination of acute myocardial infarction was demonstrated with the developed device. The ISFET device provides a platform for the multiplexed detection of different biomarkers in point-of-care testing.

## 1. Introduction

Current progress in diagnostics and medicine is highly related to fast and accurate detection of biomarkers and biomarker panels for various diseases. In this respect, the popularity of point-of-care testing (POCT) is increasing owing to the ability for simple, rapid, and inexpensive analysis [1].

Cardiovascular diseases (CVDs) are one of the major causes of death worldwide [2]. Detection of cardiac biomarkers serves for early diagnostics of CVDs, which improves clinical outcomes. Among cardiac biomarkers, cardiac troponin I (cTnI) is a well-known indicator of myocardial injury with a cut-off level of about 0.1 ng/mL [2,3] and a peak concentration reaching up to 100 ng/mL [2]. Immunoassays are dominating in laboratory detection and POCT of cTnI [4,5].

Affinity biosensors are often used as a basis for the development of POCT devices due to their high sensitivity and specificity [1]. Aptamers, short nucleic acid ligands capable of binding to a target of interest, can serve as biorecognition elements in biosensors, providing the benefits of low cost, high reproducibility, easy chemical labeling, high thermal stability, and the ability for regeneration, along with high selectivity and sensitivity [6,7]. Aptamer-based POCT systems have been developed for diagnostics of infectious diseases caused by pathogenic bacteria, viruses, fungi, and parasites and non-infectious diseases such as cancer, diabetes, and chronic respiratory and cardiovascular diseases [7,8,9,10].

Using ion-sensitive field effect transistors (ISFETs) as a transducer element of a biosensor is a convenient solution for the development of POCT devices [11,12]. The principle of ISFET sensitivity is based on the change in the surface potential of the ISFET sensing surface caused by charge distribution alteration, which leads to modulation of the drain current of the ISFET [13]. ISFETs offer the benefits of low production cost, high sensitivity, and ease of operation [12]. ISFET allows monolithic integration with signal processing circuits, enabling exceptional miniaturization and compatibility with smart portable devices [12]. Modification of an ISFET sensitive surface with a biorecognition element renders its response selective to a desired target, allowing for direct label-free detection of the analyte [14]. ISFET arrays produced within a single chip provide a basis for multiplexed biosensing [15,16], which is especially desirable for the analysis of biomarker panels. For this application, selective modification of individual ISFETs in arrays by distinct bioreceptors is required. In practice, sensing application of ISFETs is, however, restricted by sensor and temperature drift, trapped charge and capacitive attenuation, signal-to-noise ratio, and charge screening effects in electrolyte solutions [14,16]. Parallel modification of ISFETs with different bioreceptors (i.e., target-specific and non-specific aptamers) allows for the usage of ISFET/REFET differential measurements to increase the reliability of the detected signal and to compensate for noise and drift [17]. The dependence of ISFET biosensors on the charge screening effect can be reduced by using aptamers as a bioreceptor layer, since aptamers have relatively small physical size and the binding of target molecules becomes possible within the electrical double layer. Using a superimposed high-frequency voltage applied to a reference electrode (AC mode) when making ISFET measurements also helps to overcome the problem of charge screening, since in this mode the orientation of the DNA molecules at the sensing surface can be affected. This change in receptor location may contribute to a subsequent increase in the amount of charge created at the surface of the ISFET and an increase in the resulting sensitivity when detecting the DNA interactions [18,19]. In addition, the application of superimposed high-frequency voltage to a reference electrode also serves for minimizing the drift and reducing its stabilization time [14,20].

For the future practical implementation of ISFET-based biosensors to POCT, convenient and miniaturized devices for electronic readout of sensor signals are demanded. In a laboratory setting, the readout of a chip with ISFETs requires the use of a probe station and measurement equipment. Such a system is limited by the capabilities of the measuring equipment and the number of manipulators for contacting the chip pads at the probe station, which limits the scope of application and makes it difficult to measure several circuits simultaneously. Moreover, it is necessary to use various additive methods to create a reaction chamber on the surface of the chip to add liquids. A simple chip assembly with the ability to quickly install a reaction chamber and a portable device for reading and processing assembled chips can serve for both laboratory testing and POCT application. The chip assembly procedure should be compatible with the modification of ISFETs. A microfluidic system or at least a reaction chamber is required for biosensor operation. 

For POCT applications, the device should be mobile, inexpensive, and simple. To ensure the mobility, the device does not have a bulky external power source. For powering a low-power chip analog circuit, a small battery can be used to minimize signal distortion. A USB cable can be used for powering the digital circuit and for data transfer. The device must be assembled using the most available technology and parts in the world to ensure its simplicity and repairability. Device control software is required to set the operating point of the reference electrode and process the output signal from the chip to determine the original signal at the ISFET surface.

In this work, we describe the development of a portable device for simultaneous measurement of several ISFET biosensors within the same chip. The device combines ISFETs with integrated signal readout circuits with an external analog signal digitizer with DC and AC mode reference voltage options. The device assembly is compatible with chemical modification of individual ISFETs for which molecular printing technology can be used. The device performance was demonstrated with the detection of troponin I, a biomarker of acute myocardial infarction (AMI). The specific detection of troponin I was driven by a selective DNA aptamer [21]. Using the developed device, AC-mode measurements of aptamer-based biosensor were shown to be superior to the DC-based biosensor regarding signal drift, time of drift stabilization, and the minimal detected concentration of the target analyte.

## 2. Materials and Methods

### 2.1. Materials

(3-Mercaptopropyl)trimethoxysilane (MPTS) was obtained from ABRC, Karlsruhe, Germany. Ethanol, methanol, glycerol, AgNO_3_, tris(hydroxymethyl)aminomethane (tris base), tris(hydroxymethyl)aminomethane hydrochloride (tris-HCl), NaCl, and MgCl_2_ were purchased from Sigma-Aldrich, Burlington, MA, USA. SYBR Gold was obtained from Invitrogen, Carlsbad, CA, USA. Recombinant human cardiac troponin I, recombinant human myoglobin, and recombinant human N-terminal prohormone of brain natriuretic peptide (NT-proBNP) were purchased from FineTest, Wuhan, China. Pentakis(dimethylamino)tantalum(V) was obtained from DALChem, Nizhny Novgorod, Russia. 

Oligonucleotides Tro6 (5′-SH-CGCATGCCAAACGTTGCCTCATAGTTCCCTCCCCGTGTCC-3) and NBC (5′-SH-TACAAGAATCAGATTAGACCAGTTTAGAGCGCCAAATGCC-3′) were synthesized with trityl-protected SH-terminal labels by Syntol, Moscow, Russia. 

Ten mM Tris-HCl, pH 7.7, 8 mM NaCl, 1 mM MgCl_2_ buffer was used for ISFET measurements.

### 2.2. Fabrication of ISFETs with CMOS Readout Circuits

ISFETs were manufactured according to the standard CMOS 1.2 μm process. The floating gate with 30 nm Ta_2_O_5_ was formed by post-processing using the ALD method in the Fiji G2 system (Veeco, Plainview, NY, USA) at 250 °C. PDMAT and deionized H_2_O were used as precursors.

### 2.3. Characterization of ISFETs with Semiconductor Analyzer

The transmission function of the CMOS readout circuit was obtained using an Agilent B1500A semiconductor device analyzer and a Cascade PM5 probe station. The transmission function was measured at voltages Vgnd = 0 V, Vdd = 5 V, and Vg = 0–5 V. For direct contact measurements, a tungsten probe was attached to the aluminum gate of the ISFET. For measuring the transmission function in buffer, a platinum reference electrode was used.

### 2.4. Assembly of External Readout Circuit

Globally available discrete components were selected to create the device.

The 12-bit DAC MCP4922-E/SL, developed by Microchip Technology (Chandler, AZ, USA), provides a stable, high-resolution voltage output for setting the voltage at the reference electrode. The low settling time of the DAC allows for rapid voltage changes at its output, while the presence of two output channels enables the control of the reference voltage and offset voltage using a single chip. The 16-bit ADC AD7680ARMZ (manufactured by Analog Devices, Wilmington, MA, USA) features high-speed SPI interface communication and delivers high-resolution output signals with low distortion. The SPI bus operates at the closest possible frequency to the maximum of the ADC—2 MHz—which provides a maximum sampling rate of about 100 kSPS. The ADG708 (manufactured by Analog Devices, Wilmington, MA, USA) multiplexer, with its low switching time, low leakage currents, and low on-state resistance, also ensures a low level of analog signal distortion. The ATmega2560 (manufactured by Microchip Technology, Chandler, AZ, USA) microcontroller has many built-in interfaces, such as SPI and UART, with speeds that facilitate full utilization of the selected DAC and ADC. Two separate powers were used. A 9 V battery that was converted to 5 V with the low-cost, power-efficient converter and low-dropout regulator (LDO regulator) AMS 1117 (manufactured by Advanced Monolithic Systems Inc, Livermore, CA, USA) to ensure stable voltage for all analog components such as chips with ISFET, ADC, DAC, and analog multiplexers was used, along with a USB power source, to supply digital parts. Altium Designer (version 20) was utilized for connecting all these components on a single printed circuit board (PCB) and commercially fabricated in a standard PCB manufacturing facility.

### 2.5. Software Development

Software for the device was written using Python 3.12 to support GUI and several ISFET reading modes: transfer curve measurement and recording the output voltage over time. The time dependence of the output voltage can be measured with a constant offset at the reference electrode or when a discretized sinusoidal signal is applied to it. To form the latter, an array of data with sine function values at each sampling point is generated. The total number of points is determined by Equation (1):

(1) $$n=\frac{k}{f},$$

where *n* is the number of sampling points for one period of the sinusoidal signal, *k* is the number of DAC output value updates per second, and *f* is the frequency of the sinusoidal signal. Thus, the *i*-th element of the array is determined by Equation (2):

(2) $$V_{i}=A\;\sin\left(2\;\pi \;f\;t_{i}\right)+B,$$

where *A* is the amplitude of the sinusoidal signal, *B* is the voltage shift, and *t_i_* is the time after which the DAC should send the *i*-th signal relative to the beginning of the period in Equation (3):

(3) $$t_{i}=t_{i=1}+\frac{1}{n\;f},$$

An averaging function was used to increase accuracy and reduce signal fluctuations. In the transfer curve measurement mode, the number of signals for averaging is determined by the device operator, and in the output voltage recording mode, the number of approximations is equal to the maximum possible number of signals during the selected update interval. The resulting signal value is calculated using Equation (4):

(4) $$V_{res}=\frac{\sum_{i=0}^{n}V_{i}}{n},$$

Similar averaging is performed for the sinusoidal signal.

### 2.6. Formation of Reaction Chamber

Photopolymer resin Resione F69 Flexible and LCD 3D printing were used for reaction chamber creation. Reusability helps reduce plastic pollution in the environment. FreeCad (version 0.21.0) software was used to design the reaction chamber model.

### 2.7. Measurements on the Portable Device

The chip was assembled in a printed circuit board using wire bonding for three op-amp circuits in voltage follower mode. Subsequently, the reaction chamber was connected. The performance of the assembled printed circuit boards was tested on a portable device by measuring the transfer characteristics of the op-amp in follower mode at voltage on the reference electrode (*V*ref) from 0 V to 5 V and back in a buffer using a platinum reference electrode.

For DC measurements, the *V*ref was fixed at 2.4 V. In AC mode, the operating point of the reference electrode was set to DC 2.4 V with a superimposed high-frequency sine wave (0.1 Vp-p) at frequencies of 1, 10, 50, and 100 kHz.

For biosensor characterization, a portable device was used to measure the time dependence of the surface potential of a several ISFETs upon the addition of various concentrations of cardiac troponin I (cTnI), myoglobin or NT-proBNP. Before the measurements, point immobilization of Tro6 and NBC oligonucleotides was performed on the sensitive surfaces of the ISFETs. Next, a reaction chamber was installed on the printed circuit board, accommodating 15 µL of buffer. A platinum reference electrode was installed in the RC and connected to the device. One µL of analyte solution was added to the reaction chamber.

## 3. Results and Discussion

### 3.1. The ISFET with CMOS Readout Circuit

The structure of the floating gate ISFET was optimized to minimize the effects of trapped charge and capacitive attenuation. The sensing elements were integrated with initial signal processing circuits with a linear transfer function in order to minimize distortion of the original ISFET signal.

**Figure 1:**
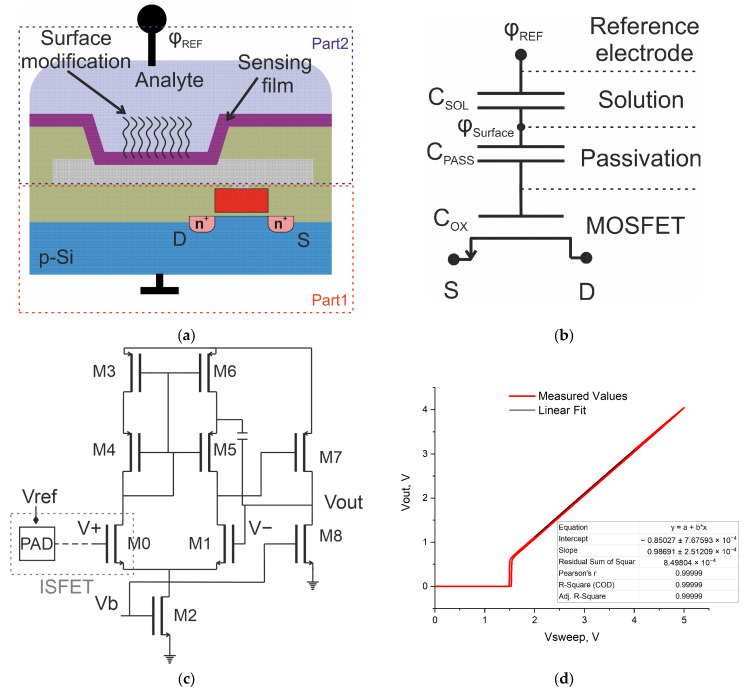
(**a**) Schematic diagram of the ISFET in CMOS technology. (**b**) ISFET equivalent circuit. *C*_SOL_ is the electrolyte capacitance, *C*_PASS_ is the passivation capacitance above the floating gate, *C*_OX_ is the gate capacitance of the MOSFET. (**c**) Electrical circuit of an ISFET with an initial signal processing scheme based on an op-amp in follower mode. M0 and M1 are input transistors of the differential pair; M2 and M8 are transistors that drive the current supply in each stage; M3, M4, M5, and M6 are transistors in a current mirror circuit with divided channel length; and M7 and M8 are transistors of the second stage of the operational amplifier, which convert the current into an output voltage. (**d**) Transfer curve of the op-amp. Gate contact is achieved using a platinum reference electrode and buffer.

ISFET structures were designed and manufactured as part of a chip using standard 1.2 μm CMOS technology to implement monolithic integration of sensitive elements with signal preprocessing circuits. These ISFETs have a floating gate and tantalum oxide sensitive surface, and are integrated with analog circuits for processing the original signal into an output voltage that can be read by an external circuit of a portable processing device. The design can be considered in two parts (Figure 1a): an opened area to the last layer of metal with a sensitive surface connected to the gate of a MOSFET (floating gate), and, separately, a MOSFET integrated into the initial signal reading circuit. As a sensitive surface of the aluminum floating gate, a 30 nm layer of Ta_2_O_5_ was used. The use of the low-temperature ALD process made it possible to integrate a chemically stable and thin high-k dielectric [22] to create an ISFET-sensitive surface on wafers with an integrated signal processing circuit manufactured via the classical CMOS IC technology. This leads to minimization of the trapped charge effects [23] due to the small number of charges/traps in a thin film of tantalum oxide, as well as to a reduction in the attenuation effect, which is discussed below. The equivalent circuit is shown in Figure 1b, where *C*_SOL_ is the electrolyte capacitance, taking into account the Gouy–Chapman and Helmholtz capacitances; *C*_PASS_ is the passivation capacitance above the floating gate (in this case, the capacitance of the sensitive surface layer); and *C*_OX_ is the gate capacitance of the MOSFET.

It is known that signals detected by ISFET due to DNA reactions can have small values [24]. Therefore, the use of initial processing circuits is required for further reading by the external circuits without distortion of the original signal. To solve this problem, approaches based on single and differential measurements, both in current and in voltage mode, are described in the literature [17]. In this work, the choice was made in favor of a voltage-mode circuit with a linear transfer function operating over a wide range of input voltages in order to use ISFETs with different threshold voltages, as well as to read the surface potential using high input impedance. Usually, voltage follower circuits are used for these tasks [17,25]. For the initial signal processing circuit, a voltage follower circuit based on an operational amplifier was chosen, since it does not require the use of processing methods to determine the initial signal of the ISFET surface potential, does not depend on environmental conditions, and has a gain factor close to unity over a wide voltage range. The follower circuit (Figure 1c) was designed on the basis of a two-stage classical operational amplifier [26]. The input transistors (M0, M1) of the differential pair and the current mirror with divided channel length (M3, M4, M5, M6) were designed using the geometric centroid method to match the transistor parameters. M7 and M8 are transistors of the second stage of the operational amplifier, which convert the current into an output voltage. M2 and M8 are transistors that drive the current supply in each stage.

Thus, an ISFET with initial signal processing was created on the basis of a 20/4 μm (W/L) transistor of a differential pair of non-inverting op-amp input (*V*_+_), with the gate connected to a 110 × 110 μm pad with a 100 × 100 μm passivation opening area. The pad size was chosen to minimize the effect of signal attenuation [27], taking into account the tantalum oxide-sensitive surface *C*_PASS_ >> *C*_OX_.

In the follower circuit, using direct contact with the floating gate, the potential on the surface of the metal pad was transmitted to the output with a gain close to unity (0.99). When contacting via liquid, a transfer curve for forward and backward sweeps was obtained, as shown in Figure 1d. The transmission ratio was 0.986.

Therefore, this design makes it possible to read the surface potential from the linear transfer function in a wide range of input voltages.

### 3.2. External Readout Circuit

**Figure 2:**
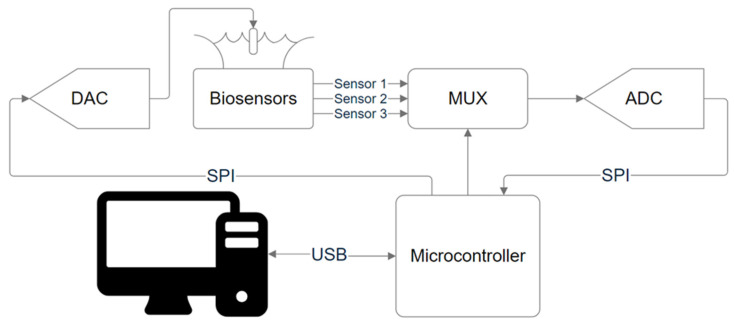
Simplified scheme of the board.

A portable device (ISFET board) was developed to read the analog signal of the sensor, which consists of the following parts: a DAC for setting the voltage at the reference electrode (MCP4922-E/SL), an analog multiplexer for selecting the ISFET reading circuit (ADG708), an ADC for reading the signal from the sensor element (AD7680), and a microcontroller (ATmega2560) responsible for controlling all components on the printed circuit board and transferring data to a computer via USB interface for subsequent processing. The SPI protocol is used to connect the DAC and ADC to the microcontroller. The SPI bus operates at the closest possible frequency to the maximum of the ADC—2 MHz—which provides a maximum sampling rate of about 100 kSPS. There is a separate power supply from a 9 V battery for the chip with ISFET and the general power supply of the device from a USB PC. The schematic diagram of the device is shown in Figure 2.

This circuit allows the signal of chips with ISFETs and the processing circuit to be measured, and solves the problem of reading several circuits simultaneously, since when using a semiconductor device analyzer and a probe station with probe positioners, there is a problem of contacting to a large number of output pads, which complicates the task of reading more than two circuits in parallel.

### 3.3. Software

The microcontroller receives a data packet at the input, which consists of the type of reading and information about active circuits. Also, depending on the type of reading, additional configuration signals are transmitted. 

To periodically executed functions such as transmitting a sine wave signal to the DAC or sending read voltage values, timers built into the microcontroller are used. Also, to increase signal integrity, all timing parameters of the components are taken into account, such as signal setup time on the DAC, ADC signal conversion time, and multiplexer switching time.

The developed software solves the problem of coordination of individual components into a unified system and using them to control operating points, obtain the transfer characteristics of sensors, receive data from sensors in real time, and process and transmit information to a PC.

### 3.4. Chip Assembly and Reaction Chamber Formation

To connect the ISFET and the initial processing circuit with the portable device, the chip was welded onto a printed circuit board with an edge card connector. This cartridge is intended for installation into an edge card slot.

**Figure 3:**
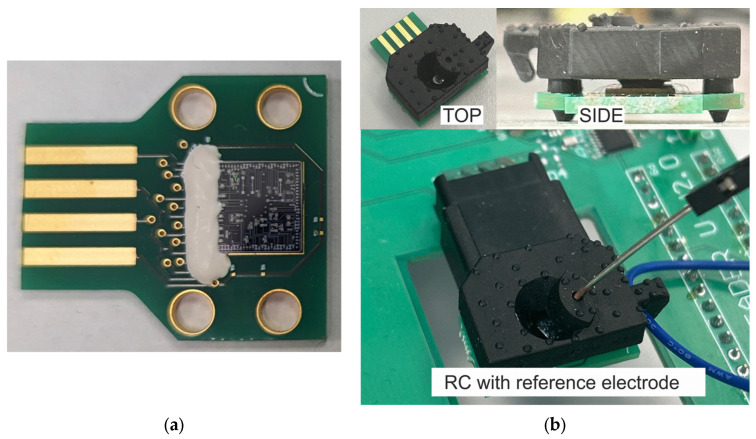
(**a**) Cartridge with a 7 × 7 mm chip for size comparison; (**b**) reaction chamber (RC) latched onto a printed circuit board with a chip and a reference electrode connected to a portable stand.

This design has the following advantages: the absence of a complex chip packaging operation, and easy and fast cartridge replacement. An image of the cartridge with a 7 × 7 mm chip for size comparison is shown in Figure 3a.

To supply an analyte to the surface of the chip and to set the reference voltage, a removable reaction chamber was designed. It is easily combined with cartridges and provides a sealed connection to the chip.

The chamber is made of Resione A 69 Flexible photopolymer resin by LCD 3D printing. After latching onto the printed circuit board, it forms an insulated reservoir for liquid contact with the surface of the chip (Figure 3).

In addition, the possibility of installing an external reference electrode was built into the reaction chamber. In Figure 3b, the possibility of such an installation is demonstrated.

### 3.5. Device Testing

#### 3.5.1. Characterization of ISFETs with Readout Circuits on Portable Device

The chip was assembled into a printed circuit board using wire bonding for three op-amp circuits in voltage follower mode. Subsequently, the reaction chamber was connected (Figure 3b).

**Figure 4:**
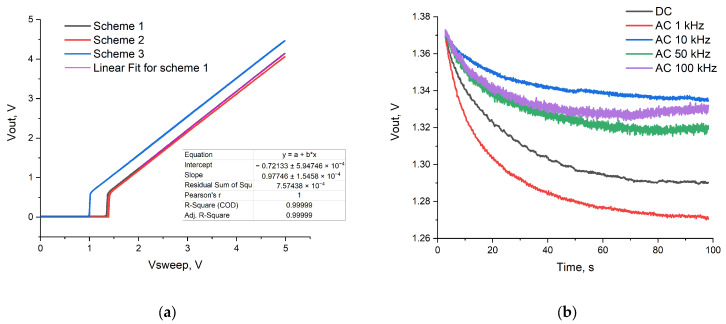
(**a**) The transfer curve of three op-amp circuits with a liquid buffer contact and a platinum reference electrode, measured on a portable device; (**b**) time dependence of baseline drift for DC and AC (1, 10, 50, 100 kHz 0.1Vp-p) modes.

The performance of the assembled printed circuit boards was tested on a portable device by measuring the transfer characteristics of the op-amp in follower mode from 0 V to 5 V and back in a buffer using a platinum reference electrode (Figure 4a). The gain was found to be about 0.977.

The baseline drift was compared at DC and AC sine waves (1, 10, 50, and 100 kHz 0.1Vp-p) reference voltages using a platinum reference electrode in a buffer solution (10 mM Tris-HCl, pH 7.7, 8 mM NaCl, 1 mM MgCl_2_). The use of AC reference voltage reduced the drift and stabilization time in comparison with the DC mode (Figure 4b). In AC mode, baseline drift stabilization was achieved with a lower voltage decrease at a frequency of 10 kHz and superimposed high-frequency voltage of 0.1 Vp-p. This ensured a smaller drop from the reference voltage, which was very important so as not to leave the output range during measurements.

#### 3.5.2. Sensor Modification with Bioreceptor

The main requirement for the chip assembly stage is compatibility with the process of immobilization of the chip surface using the molecular printing system Nano eNabler (Bioforce Nanosciences, Ames, IA, USA). For this, the surface of the chip must be completely open after assembly, which was achieved via reaction chamber (RC) design.

**Figure 5:**
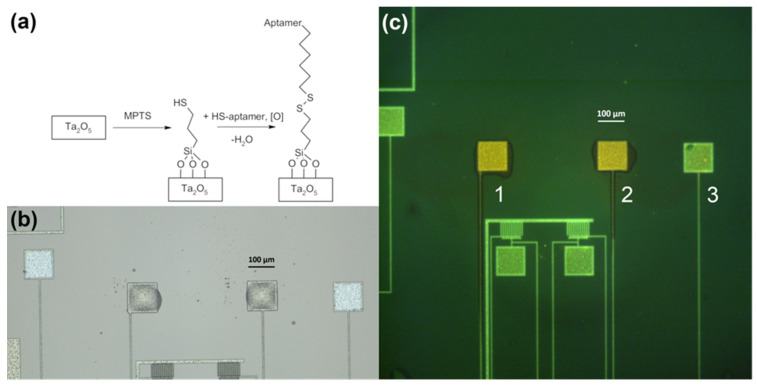
Modification of the sensor surface. (**a**) Immobilization chemistry. (**b**) Sensor pads covered with oligonucleotide solution. (**c**) Modified (1, 2) and non-modified (3) sensor pad fluorescence in the green channel after SYBR gold staining.

For the immobilization stage, the following process compatible with the PCB technology discussed above was developed. A DNA aptamer specific to troponin I (Tro6, [21]) was covalently attached to the sensitive surface of an individual sensor pad. Another oligonucleotide that served as a non-binding control (NBC) was immobilized on another pad in parallel. Molecular printing technology was used to perform modification of individual sensors. Molecular printing allows for precise and reproducible dispensing of very small liquid drops onto a surface with a robotized device under microscopic control [28]. Disulfide bonding was used for covalent immobilization (Figure 5a). The drops of SH-labeled oligonucleotides solutions were dispensed onto the sulfhydryl activated sensor pads (Figure 5b). Immobilization was confirmed with fluorescent microscopy (Figure 5c).

The described immobilization procedure based on molecular printing was incorporated into the cartridge assembly process due to the removable reaction chamber. Other chemistries can be used for surface modification with this procedure as well. The strategy allows for individual modification of the desired quantity of sensor pads with distinct bioreceptors serving for the realization of differential measurements and multiplexed analysis.

#### 3.5.3. Biosensor Demonstration

**Figure 6:**
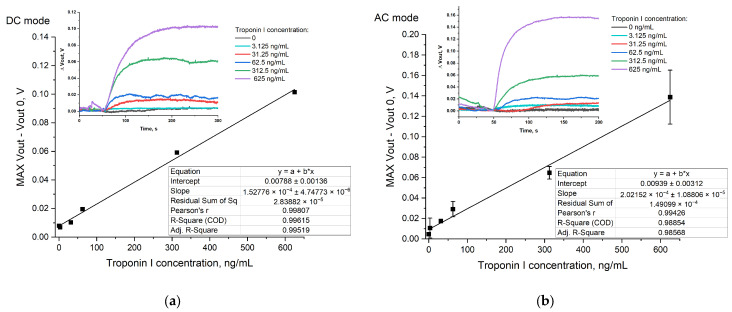
Sensor response upon addition of troponin I at different concentrations and signal plot against troponin I concentration: (**a**) DC mode (a single measurement was taken for each point); (**b**) AC mode (each point was measured in duplicate).

The response of the sensors modified with Tro6 aptamer upon different concentrations of cTnI was analyzed in both DC and AC modes. Sensor pads 1 and 2 were modified with Tro6 aptamer and non-binding oligonucleotide, respectively. Sensor pad 3 was not modified. The reaction chamber was filled with the buffer (10 mM Tris-HCl, pH 7.7, 8 mM NaCl, 1 mM MgCl_2_) and a Pt reference electrode was incorporated. The intensity of the biosensor response increased alongside the cTnI concentration (Figure 6). 

In DC mode, the response was linear in the concentration range of 31.25 ng/mL–625 ng/mL (130 pM–26.125 µM, Figure 6a). The limit of detection (LoD) was calculated to be 15.77 ng/mL.

In AC mode, the operating point of the reference electrode was set to the value (2.4 V and 0.1 Vp-p superimposed high-frequency voltage). The obtained concentration curve in AC was characterized by lower LoD (3.27 ng/mL), which allowed for the detection of AMI as a serum concentration of troponin I under AMI is about 30 ng/mL [2,3]. The increase in sensitivity and LoD in AC mode compared to DC can be explained by the disposition of the aptamer chain with respect to the sensor surface. In the aptasensor, the ISFET detects the charge redistribution inside the Debye length that occurs upon aptamer–target interaction. Namely, upon binding with troponin I, the negatively charged aptamer molecule moves away from the surface, reducing the effective charge inside the Debye length and resulting in a change in the sensor signal. The applied alternating voltage can affect the orientation of DNA on the surface, which can therefore affect the number of molecules that can generate a charge change within the Debye length. Thus, AC measurements serve for minimization of the sensor drift and time required for drift stabilization, and allow for the minimal target concentration that can be detected by the ISFET aptasensor to be decreased.

**Figure 7:**
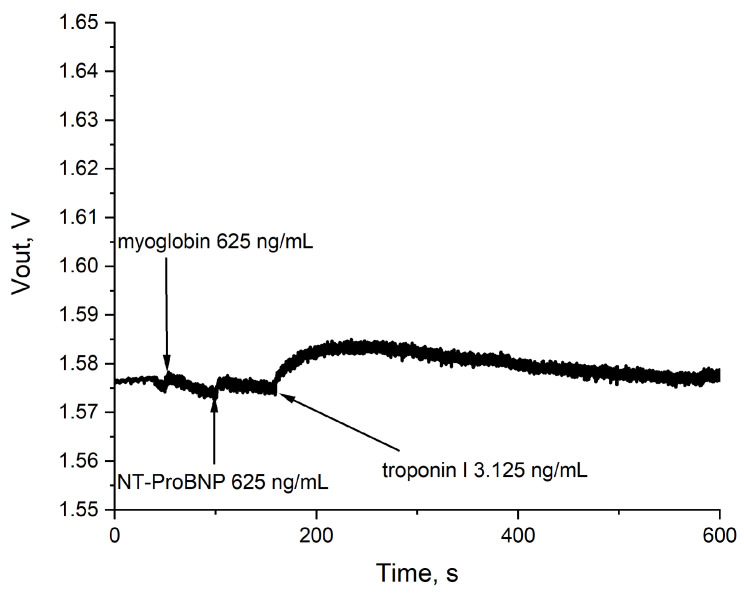
Specificity of the sensor signal. Myoglobin (625 ng/mL), NT-proBNP (625 ng/mL), and troponin I (3.125 ng/mL) were sequentially added at 50, 100, and 150 s of the measurement, respectively.

No response of the aptamer-modified sensor in 10 kHz 0.1 Vp-p AC mode was detected upon addition of myoglobin and N-terminal prohormone of brain natriuretic peptide (NT-proBNP) at 625 n/mL concentration, confirming aptasensor specificity (Figure 7). Detection of these cardiac biomarkers along with Troponin I is of interest, and interference should be avoided [29,30,31]. The tested concentration of NT-proBNP and myoglobin were in range with peak pathologic concentrations of 66 ng/mL [32] and 474 ng/mL [33], respectively.

Biosensor characteristics of other FET-based aptasensors for the detection of troponin I are summarized in Table 1. The LoD and linear range of these biosensors differ significantly and depend on the sensor type, specific aptamer sequence, and even immobilization chemistry [34].

#### 3.5.4. Real Sample Measurements

**Figure 8:**
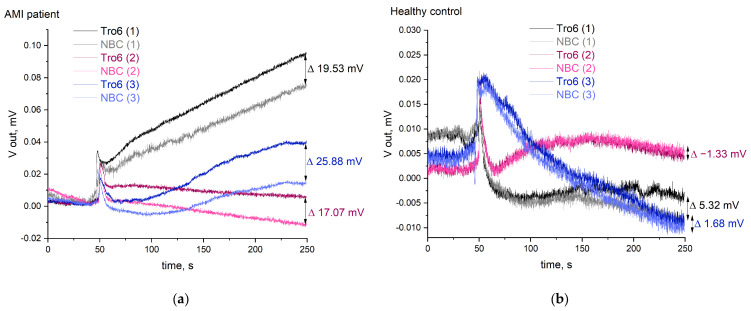
Real sample measurements with the developed device: (**a**) analysis of serum of an AMI patient; (**b**) analysis of serum of a healthy person.

Serum of an AMI patient with 23.497 ng/mL troponin I detected in semiquantitative clinical laboratory assay and serum of a healthy person with no troponin I detected were analyzed on the developed device in AC mode (Figure 8). Three measurements were performed for each sample. For the same sample, the time curves differed from each other, and the values of signal intensity—calculated as (*V*_out max_–*V*_out 0_), like for the calibration in buffer—varied strongly. These signal alterations were not observed for troponin I in buffer and can be explained by the diffusion of the proteins and salts of the serum towards the sensor surface. However, this effect could be efficiently compensated for using ISFET/REFET measurements. Within 200 s after sample addition, the difference in surface potential between aptamer- and non-binding oligonucleotide sensors reached 20.8 ± 4.6 mV for the serum of the AMI patient (Figure 8a) and reached 1.9 ± 1.3 mV for the serum of the healthy person (Figure 8b). The difference between these values was statistically significant (*p* < 0.01). So far, the developed device operated in AC mode is potent for qualitative discrimination of AMI. Recalibration with troponin I standards in serum can provide a basis for further quantitative assays.

## 4. Conclusions

Ion-sensitive field effect transistors in conjugation with aptamers offer a promising platform for POCT of biomarkers and biomarker panels. In this study, a portable device using an ISFET aptasensor for detection of a protein biomarker was developed. ISFETs integrated with CMOS readout circuits were designed and manufactured to enable the readout of the surface potential of ISFET sensor pads with a linear transfer function over a wide range of input voltages. A PC-operated portable ISFET board enabled simultaneous measurement of the various ISFETs with CMOS readout circuits. The developed device supports DC and AC measurement modes. The chip assembly with a removable reaction chamber allowed for modification of individual sensor pads with different DNA aptamers using molecular-printing technology. Selective modification of ISFETs offers differential measurement options and opens the possibility for multiplexed sensing. A wide range of biomarkers can be detected with FET-based aptasensors [43,44], and there is a rationale to combine the detection of troponin I with other cardiac biomarkers such as NT-proBNP, fibrinogen, copeptin, creatine kinase, myoglobin, and the markers of inflammation (C-reactive protein, interleukine-6, interleukine-12) to develop multi-marker panels needed for clinical applications [29,31,45,46,47]. To demonstrate the operational performance of the device, ISFET sensor pads were modified with a DNA aptamer for troponin I, which is a well-known biomarker of acute myocardial infarction, and with non-binding DNA, which served as a control for differential measurements. Comparison of DC and AC methods for setting the reference point on the reference electrode revealed that AC mode decreased ISFET drift over time and helped to reduce the minimum concentration of troponin I detected by the aptasensor. The developed device allows for qualitative discrimination of AMI. Further studies are required to achieve quantitative detection of troponin I in serum samples. As a future research direction, the study of troponin I detection from saliva would bring the device closer to POCT application. Detection from saliva is non-invasive and more affordable for patients, but troponin I levels in saliva are considered to be lower compared to those in serum. The bottleneck of the described methodology is the low throughput of hand-held chip assembly and the chemical modification of ISFETs, both of which can hamper mass production of the device.

## Figures and Tables

**Table 1:** Characteristics of FET-based aptasensors for troponin I detection.

Sensor Type	Aptamer Used	LoD, ng/mL	Linear Range, ng/mL	Reference
Polysilicon FET	55-nt DNA, no sequence provided	0.1	0.1–1000	[35]
Silicon nanowire FET	79 nt DNA aptamer, no sequence provided	1	1–1000	[36]
Silicon nanowire FET	79 nt DNA aptamer, 5′-GCCTGTTGTGAGCCTCCTAACTACATGTTCTCAGGGTTGAGGCTGGATGGCGATGGTGGCATGCTTATTCTTGTCTCCC-3′	0.0239	10–1000	[37]
Silicon nanowire FET	Tro6, 40 nt DNA, 5′-CGCATGCCAAACGTTGCCTCATAGTTCCCTCCCCGTGTCC-3′	0.002	0.002–0.008	[34]
Ta_2_O_5_-gated FET	Tro6, 40 nt DNA, 5′-CGCATGCCAAACGTTGCCTCATAGTTCCCTCCCCGTGTCC-3′	3.27	3.27–625	This work
Carbon nanotube network FET decorated with gold nanoparticles	Tro6, 40 nt DNA, 5′-CGCATGCCAAACGTTGCCTCATAGTTCCCTCCCCGTGTCC-3′	2.4	2.4–2400	[38]
Graphene-based FET	Tro4, 40 nt DNA, 5′-CGTGCAGTACGCCAACCTTTCTCATGCGCTGCCCCTCTTA-3′	0.00334	0.01–0.50	[39]
Graphene-based FET	Tro4, 40 nt DNA, 5′-CGTGCAGTACGCCAACCTTTCTCATGCGCTGCCCCTCTTA-3′	0.0033	0.0033–0.2	[40]
Magnetic graphene FET	Tro4, 40 nt DNA, 5′-CGTGCAGTACGCCAACCTTTCTCATGCGCTGCCCCTCTTA-3′	0.239	0.239–23,900	[41]
Metallic nanowire FET	Tro4, 40 nt DNA, 5′-CGTGCAGTACGCCAACCTTTCTCATGCGCTGCCCCTCTTA-3′ data	0.000013	0.0001–10	[42]

## Data Availability

Data are contained within the article.
